# Graphic Warning Labels Affect Hypothetical Cigarette Purchasing Behavior among Smokers Living with HIV

**DOI:** 10.3390/ijerph16183380

**Published:** 2019-09-12

**Authors:** Lauren R. Pacek, Meredith S. Berry, Olga Rass, Melissa Mercincavage, F. Joseph McClernon, Matthew W. Johnson

**Affiliations:** 1 Department of Psychiatry and Behavioral Sciences, Duke University School of Medicine, Durham, NC 27705, USA; francis.mcclernon@duke.edu; 2 Department of Psychiatry and Behavioral Sciences, Johns Hopkins University School of Medicine, Baltimore, MD 21224, USA; mberry@ufl.edu (M.S.B.); olga.rass@gmail.com (O.R.); mwj@jhu.edu (M.W.J.); 3 Department of Health Education and Behavior & Department of Psychology, University of Florida, Gainesville, FL 32611, USA; 4 Department of Psychiatry, University of Pennsylvania, Philadelphia, PA 19104, USA; melmer@pennmedicine.upenn.edu

**Keywords:** graphic warning labels, pictorial warning labels, tobacco control, tobacco, smoking, HIV, comorbidity, behavioral economics

## Abstract

Cigarette pack graphic warning labels (GWLs) are associated with increased knowledge of tobacco-related harms; scant research has evaluated their effects on behavior among vulnerable populations. We used a behavioral economic approach to measure the effects of GWLs and price on hypothetical cigarette purchasing behavior among HIV-positive smokers. Participants (n = 222) completed a cigarette valuation task by making hypothetical choices between GWL cigarette packs at a fixed price ($7.00) and text-only warning label cigarette packs at increasing prices ($3.50 to $14.00; $0.25 increments). More than one-quarter (28.8%) of participants paid more to avoid GWLs. The remaining participants’ purchasing decisions appear to have been driven by price: 69.8% of participants chose the cheaper pack. Across all participants, overall monetary choice value observed for GWL cigarette packs (mean = $7.75) was greater than if choice was driven exclusively by price ($7.00). Most (87.4%) preferred the text-only warning label when GWL and text-only cigarette packs were equally priced. Correlation analysis indicated GWL pack preference was associated with agreement with statements that GWLs would stop individuals from having a cigarette or facilitate thoughts about quitting. These data suggest that GWLs may influence some HIV-positive smokers in such a way that they are willing to pay more to avoid seeing GWLs.

## 1. Introduction

Health warnings on tobacco product packaging communicate health risks associated with tobacco use and can serve as a population-level smoking cessation intervention. Warning labels that are larger and include graphics in addition to textual messages are associated with a greater impact than smaller, text-only labels [1,2,3,4]. These pictorial or graphic warning labels (GWLs) are associated with increased knowledge about tobacco-related harms and decreased experimentation among nonsmokers, as well as deterred smoking, increased intentions and attempts to quit, and reduced relapse among smokers [1,3,5]. Observational studies have found increased cessation in populations where GWLs have been implemented [6,7]. Further, a large randomized controlled trial found that smokers who used cigarette packs affixed with GWLs prominently displayed on the front of the package for four weeks had greater odds of abstinence compared to those given cigarette packs with text-only warnings affixed to the side [8].

While these studies suggest GWLs offer potential benefit to public health, the attempts of the U.S. Food and Drug Administration (FDA) to require GWLs on cigarette packs were delayed in 2012 by court challenges from the tobacco industry [9]. Recently, however, a federal judge ordered the FDA to expedite GWL implementation [10]. Additional experimental studies that determine a link between the use of GWLs and behavior would strengthen the evidence base demonstrating the efficacy of GWLs in reducing smoking behaviors.

Vulnerable populations may receive further benefit from the GWLs on cigarette packs. Persons living with human immunodeficiency virus (HIV) bear a disproportionate burden in terms of smoking prevalence and smoking-related health sequelae [11,12,13]. Specifically, persons living with HIV currently lose a greater number of life years to smoking than to HIV (15 versus 3 years) [13]: 24% of all deaths among persons on antiretroviral (ARV) medications are attributable to tobacco use [14], and smoking is associated with poor adherence to and decreased effectiveness of ARV medications [15,16,17]. Notably, a significant proportion of persons with HIV are estimated to experience low health literacy (i.e., the ability for individuals to obtain, process, and understand health information and services in order to make decisions) [18,19,20,21]. Given that prior work has demonstrated that GWLs are perceived as being more credible and effective by low health literacy participants [22] and that GWLs are more likely to increase intentions to quit smoking among low health literacy participants [23], GWLs may be a particularly effective intervention for persons living with HIV.

The present study sought to evaluate the behavioral impact of GWLs on decisions to purchase packs of cigarettes in a novel cigarette valuation task among smokers living with HIV. We developed a task—with similarities to previous tasks used to determine hypothetical tobacco/nicotine product purchase decisions [24,25,26]—in which participants made a series of choices between a cigarette pack with a GWL at a fixed price and a cigarette pack with a text-only warning label at increasing prices. The task was designed to permit the determination of a specific monetary value as a metric for the potential reduction in reinforcing value caused by the presence of a GWL. To our knowledge, a study producing this valuable outcome has never been published, either among the general population of smokers or among smokers living with HIV. 

## 2. Methods

### 2.1. Data Source

Data were from an online survey that recruited U.S. cigarette smokers living with HIV. Methods for this survey have been described elsewhere [27,28,29], but briefly: Participants were recruited from Amazon Mechanical Turk (mTurk), a crowdsourcing platform that has been used to collect data on attitudes, perceptions, and behaviors related to tobacco use [24,30,31,32,33]. Participants were eligible if they were U.S. residents, had a diagnosis of HIV, were established, current smokers (≥100 cigarettes lifetime, ≥1 cigarette in the past month), and were ≥18 years of age. Data were collected from 16 March–14 May 2015. Participation was voluntary, anonymous, and participants were paid $1 upon completion. Study procedures were approved by the Institutional Review Board at Johns Hopkins University (protocol approval #NA00084986). To improve data quality, participants were asked (a) if they took their time in completing the survey; (b) if their data should be retained; and (c) if they experienced any computer problems during the survey. Individuals indicating problematic data were compensated, but their data were excluded from analysis.

Two hundred and seventy-eight participants began the survey. Of these, 21 did not finish the survey, 9 were removed for answering “not at all” to the question “do you currently smoke cigarettes?”, 3 were removed due to computer issues during the survey (e.g., computer froze or restarted during the survey), and 23 were removed for nonsystematic discounting (as described below). In total, data from 222 participants were included in subsequent analyses. 

### 2.2. Sociodemographic Variables

Sociodemographic measures included sex, age (continuous), race (White, Black/African–American, Asian, more than one race, Native American, Pacific Islander, other), ethnicity, and educational attainment (no high school diploma, high school diploma or equivalent (GED), some college education, trade/technical/vocational training after high school, associate’s degree, bachelor’s degree, master’s degree, professional/doctorate degree). 

### 2.3. Smoking Characteristic Variables

Participants completed smoking history questions, including past month smoking rate (cigarettes per day (CPD) and number of days smoked in the past 30), age of smoking initiation, time to first cigarette (TTFC), and interest in quitting or cutting down on their smoking. Participants also completed the contemplation ladder, a 0–10 scale assessing smokers’ thoughts on quitting (0 = no thought of quitting, 10 = taking action to quit). The Heaviness of Smoking Index (HSI) [34] was calculated from CPD and TTFC variables; scores were categorized as low (0–1), moderate (2–4), and high (5–6) dependence [35]. Participants also completed questions regarding their HIV diagnosis and treatment regimen, cessation attempts and intent, and knowledge and beliefs about smoking and health that have been reported previously [27,28,29]. 

### 2.4. Cigarette Valuation Task

**Figure 1:**
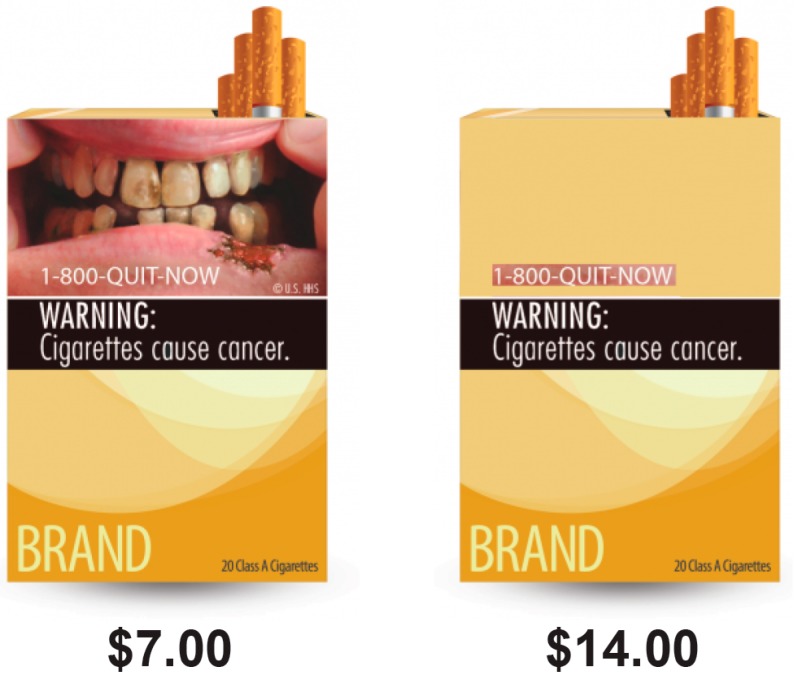
Example question from the hypothetical cigarette valuation task.

Participants were randomly assigned to view one type of GWL cigarette pack (see Supplementary Figure S1). Throughout the task, participants chose between either the GWL cigarette pack or the associated text-only version of the cigarette pack. There were 43 price points for the text-only pack (from $3.50 to $14.00 in $0.25 increments), whereas, the prices of the graphic plus text pack were fixed (Figure 1). That is, in each of the 43 individual trials, participants were asked to choose between purchasing a pack of cigarettes with one of the 9 FDA-proposed text plus GWLs, or a pack of cigarettes with a text-only warning. Participants who reported not having or living with children and no intent to have children (i.e., biological, adopted, stepchildren, or foster children) were not assigned to the following GWL conditions: (1) “Tobacco smoke can harm your children”, or (2) “smoking during pregnancy can harm your baby”. Participants were given the following instructions: Each hypothetical question represented a new day; their current financial circumstances should be considered; the cigarettes available were their preferred brand, but the packaging was changed; the products specified were the only nicotine/tobacco product available over the next 24 h; and the cigarettes needed to be consumed within 24 h and could not be saved, given away, or sold.

### 2.5. Reactions to GWLs

To measure reactions to the GWL seen during the hypothetical purchase task, participants rated the following statements on a 5-point Likert scale (definitely false, somewhat false, neither true nor false, somewhat true, definitely true): (1) The message in this image is _________; (2) seeing this image on my cigarette pack would stop me from having a cigarette when I am about to smoke one; (3) seeing this image on my cigarette pack would make me think about the risks of smoking to my own health; (4) seeing this image on my cigarette pack would make me think about the risks of smoking to the health of others; and (5) seeing this image on my cigarette pack would make me think about quitting smoking. 

### 2.6. Statistical Analysis

#### 2.6.1. Orderliness of Data

Participant data were considered nonsystematic and excluded listwise from analysis if he/she “switched” from a GWL pack of cigarettes to a text-only warning label pack of cigarettes or vice versa more than a single time throughout the choice procedure (in which the cost of text-only warning label cigarette packs increased in $0.25 increments from $3.50 to $14.00, and GWL cigarette packs remained constant at $7.00). In other words, more than a single “switch point” was considered non-systematic (n = 23 participants were excluded for this reason). This exclusion criterion assumed that choices across the task were not random and remained logically and internally consistent. There were no systematic differences between the participants eliminated compared with those who were not on any demographic or smoking characteristic variable examined (including age, sex, race, ethnicity, marital status, income, age at first cigarette, or responses to the images (e.g., “seeing this image made me want to quit smoking”) and interest in quitting or interest in cutting down on smoking).

#### 2.6.2. Data Characterization and Analyses

One measure of choice was used to characterize the remaining data sets: The dollar value at which the text-only cigarette pack was subjectively equal to the GWL cigarette pack (i.e., the “switch” point) for each participant. Specifically, the switch point was defined as the greatest dollar value of text-only cigarette pack selected prior to “switching” to the GWL cigarette packs. For some participants, no “switch” occurred (i.e., participants exclusively chose cigarette packs with the same type of warning label). In cases where the participant exclusively chose the text-only cigarette pack (which consistently increased in price by increments of $0.25), the value of their final choice was used ($14.00). In cases where the participant exclusively chose the GWL cigarette pack (which remained constant at $7.00) a value of $3.25 was used. These data were not normally distributed and therefore non-parametric statistics were used. 

Using the methods described above to determine the switch points as the primary dependent measure for each participant, our primary analyses were designed to characterize the effect of GWLs on participant switch points. In other words, we evaluated how GWLs versus text-only labels influenced choice and willingness to pay for cigarettes. To evaluate this, we first characterized the overall sample, and then the percentage of individuals who appear to have been exclusively driven by price (i.e., only purchased the most inexpensive cigarette pack across all choices), versus those who were willing to pay more for text-only cigarette packs. We also examined the distribution using descriptive statistics of these distinct groups. Second, switch points across the different GWLs were compared using a Kruskal-Wallis H test to evaluate if GWL type (e.g., warning regarding lung disease versus warning regarding addictiveness of cigarettes) would result in significantly different switch points. 

Third, a Wilcoxon signed-rank test was used (due to non-normal distribution of switch points) to test whether switch points were significantly different from the hypothesized value of $7.00 (the value of the GWL cigarette pack and the switch point value indicating choice was driven by price). A significant difference between the value of $7.00 and observed switch points would indicate that choice and resulting switch points were not exclusively driven by price (i.e., participants did not solely choose the cheapest option), and that participants were willing to pay more for cigarette packs that did not display GWLs. 

Finally, to determine relations between switch points and participant demographic and smoking characteristics, Spearman’s correlations were applied (due to the non-normal distribution of switch points). Based on previous literature and predictions that graphic warning labels may have differential effects depending on age [36], and may be more credible among low health literacy populations [22], age, education, and income, correlations between switch points and sociodemographic characteristics were determined. Based on previous literature showing that smoking characteristics may be related to graphic warning label responses and perceptions [37], we also determined correlations between switch points and cigarettes smoked per day, quit ladder score, interest in quitting, Heaviness of Smoking Index (HSI) and participant responses to the questions “seeing this image on my cigarette pack would stop me from having a cigarette when I am about to smoke one” as well as “seeing this image on my cigarette pack would make me think about quitting smoking” (answer options: Definitely false = 1; somewhat false = 2; neither true nor false = 3; somewhat true = 4; definitely true = 5), as well as sex, and having children or planning to have children. Analyses were performed using GraphPad Prism v.7.0 [38] and SPSS v.21.0 [39]. Statistical tests were considered significant at α = 0.05. 

## 3. Results

### 3.1. Sociodemographic and Smoking Characteristic Data 

The far left column of the results portion of Table 1 displays the percentages and means for the entire sample. Overall, the sample was predominantly male and white, and the average age was 28.7 (SD = 7.7) years old (Table 1). On average, participants smoked 21.3 (SD = 9.8) days within the past month, smoked their first cigarette at age 15.4 (SD = 3.3), and had a mean contemplation ladder score of 4.8 (SD = 3.1). For comparison purposes, the results in Table 1 are also demarcated by participants who chose to pay more than $7.00 to avoid GWL packs of cigarettes (center results column) compared to those who paid less than or equal to $7.00 (far right column).

### 3.2. Data Characterization and Analyses

Between 21 and 29 participants were assigned to each image condition. The overall sample median switch point was $7.00 (minimum = $3.25, maximum = $14.00). When the choice for the GWL versus text-only cigarette packs were equivalent in price (both cost $7.00), 87.4% of the sample chose the text-only label. The majority of participants (69.8% of the sample, median switch point, minimum and maximum = $7.00) also consistently chose the inexpensive option, regardless of text-only or GWL cigarette packs. However, a substantial number of participants were willing to pay more to avoid the GWLs, opting for text-only warning label cigarette packs at higher prices (n = 64; 28.8% of the sample; median switch point $8.75; minimum = $7.25, maximum = $14.00). That is, these participants were willing to pay more for text-only warning label cigarettes, even when a cheaper pack of GWL cigarettes was available. A small minority of participants chose the GWL cigarette packs exclusively (n = 5; all switch points coded as $3.25) or switched to the GWL cigarette packs while the text-only warning label packs were cheaper (n = 3; switch points of $3.75, $5.75, and $6.25). 

In order to test for potential differences in the effect of differing GWLs on switch points, a Kruskal-Wallis H test (used for non-normal distributions) was used to compare the rank value of switch points across each of the 9 different GWL conditions. The independent samples Kruskal-Wallis H test revealed an overall significant difference in switch points across the different graphic warning labels, *χ*^2 ^(8, *N =* 222) = 26.97, *p <* 0.01. Applying a Bonferroni correction to account for family-wise error rate, follow-up post hoc pairwise comparisons revealed that the graphic warning label displaying decaying teeth and a message stating “cigarettes cause cancer”, as well as a drawing of an infant with a message stating “smoking during pregnancy can harm your baby” resulted in significantly higher switch points (i.e., participants were willing to pay more to avoid that label) relative to the GWL displaying a man wearing an “I quit” t-shirt next to the message “quitting smoking now greatly reduces serious risks to your health” (*p <* 0.001 and *p* < 0.04, respectively). No other single comparison across graphic warning labels revealed significant differences in switch point values (all other *p’s >* 0.051).

A Wilcoxon signed-rank test was used (due to non-normal distributions) to test whether switch points were significantly different from the hypothesized value of $7.00 (i.e., the value of the GWL cigarette pack and the switch point value indicating choice was driven by price). Switch point ranks were significantly higher in the observed data compared to the hypothesized value of $7.00 (Z = −5.31, *p <* 0.001). These results suggest that participants were willing to pay more for cigarette packs that did not display GWLs.

Spearman’s correlations for the associations between switch points and participant sociodemographic and smoking characteristics are presented in Table 2. Strong significant positive correlations were observed between switch points and agreement with the statement “seeing this image on my cigarette pack would make me think about the risks of smoking to my own health”, as well as “seeing this image on my cigarette pack would make me think about quitting smoking.” A strong significant positive correlation was also revealed between participant Contemplation Ladder score and agreement with the statements “seeing this image on my cigarette pack would stop me from having a cigarette”, “seeing this image on my cigarette pack would make me think about the risks of smoking to my own health”, “seeing this image on my cigarette pack would make me think about the risks of smoking to the health of others”, and “seeing this image on my cigarette pack would make me think about quitting smoking.” Income and education were strongly positively correlated; however, income or education was not significantly correlated with switch point. No significant correlations were observed between demographic variables and switch points. 

## 4. Discussion

The present study used a novel cigarette valuation task to examine the impact of GWLs, compared to text-only warning labels, on cigarette packs on hypothetical cigarette purchasing behavior among HIV-positive cigarette smokers. Results indicate that the majority of participants’ purchasing decisions were driven exclusively by price, with most participants consistently choosing the cheaper pack of cigarettes. However, when GWL and text-only cigarette packs were equal in price, the vast majority of participants chose the text-only warning label. Moreover, more than one quarter of participants were willing to pay more to avoid the GWLs. This resulted in the choice of significantly higher priced text-only cigarette packs (i.e., higher switch points) for the overall sample than the standard price of the GWL cigarette packs. These findings are consistent with what has been observed in the literature: GWLs produce negative emotional responses [40]—and do so to a greater degree than non-GWLs [41]—as well as result in attempts to avoid viewing GWLs [40]. 

Additionally, we identified strong and significant positive correlations between switch points and agreement with the statement “seeing this image on my cigarette pack would stop me from having a cigarette” as well as the statement “seeing this image on my cigarette pack would make me think about quitting smoking.” Consistent with these findings, Hammond and colleagues [40] found that smokers who reported greater negative emotional reactions to GWLs were more likely to quit, attempt to quit, or reduce their smoking at follow-up. These findings are also consistent with the broader literature concerning the relation between risk perceptions of cigarette smoking and smoking behaviors themselves. Smoking risk perceptions are associated with smoking status and interest in quitting [42,43], and are predictive of quit attempts and sustained quitting [44,45]. Concerns about the health risks of smoking are also the most common motivation to quit, as reported by current and former smokers [44,46,47,48].

There are several limitations associated with the present study that should be considered. First, given the online nature of the sample (i.e., mTurk), generalizability of findings to persons living with HIV more broadly may be limited. Additionally, this study relied on decision making in hypothetical scenarios. However, it should be noted that hypothetical cigarette purchasing tasks have been shown to be correlated with actual smoking behaviors, including cessation outcomes [49,50]. Regardless, future work could be conducted in laboratory or clinical trials contexts in order to evaluate actual purchasing behavior. Though several studies support the validity and reliability of hypothetical purchasing tasks for cigarettes [51,52,53], the task in the present study differed from typical and validated cigarette purchase tasks in its assessment of pack purchases rather than single cigarettes and addition of GWLs as a manipulation. As a result, it is possible that the validity and reliability of traditional purchase tasks does not extend to the present results. Future work should evaluate actual purchasing/cigarette consumption behavior in response to GWL manipulations, in both acute studies as well as using longitudinal study designs. It is also important to note that the correlations presented are two variable associations. Future more complex analyses with larger sample sizes might begin to address the influence of multiple variables simultaneously on switch points. An additional limitation is that the present study did not collect explicit information regarding participants’ level of health literacy. Future research would benefit from examining whether this construct impacts hypothetical cigarette pack purchasing behavior. 

## 5. Conclusions

These limitations notwithstanding, to our knowledge, this work represents the first to examine the impact of GWLs versus text-only warning labels on cigarette pack valuation and purchasing behavior generally and specifically among cigarette smokers living with HIV. Findings indicate that, while most participants were driven exclusively by price—selecting the less expensive cigarette pack regardless of appearance—a significant proportion of the sample (i.e., more than one-quarter) were willing to pay more money in order to avoid purchasing the GWL cigarette packs in favor of the text-only warning label. Participants’ selection of cigarette packs was also significantly associated with statements indicating that viewing GWLs would induce thoughts of quitting smoking and would stop participants from having a cigarette. Collectively, our data suggest that the presence of GWLs on cigarette packs may influence some HIV-positive smokers—a vulnerable population of smokers who are disproportionately burdened by the prevalence and associated health sequelae of tobacco use—in such a way that they are willing to pay more for cigarettes in order to avoid viewing GWLs. Ultimately, the presence of GWLs should increase thinking about the health effects of smoking and increase motivation to quit.

## Figures and Tables

**Table 1:** Participant characteristics (n = 222).

Characteristic	% (n)	% (n) > $7.00	% (n) ≤ $7.00
Demographic Characteristics			
Female sex	35.1 (78)	37.5 (24)	34.1 (54)
Age, mean (SD)	28.7 (7.7)	28.1 (7.5)	29.1 (7.8)
Race			
White	75.2 (167)	75.0 (48)	75.3 (119)
Black/African–American	11.2 (25)	10.9 (7)	11.4 (18)
Asian	7.2 (16)	3.1 (2)	8.9 (14)
More than one race	4.0 (9)	7.8 (5)	2.5 (4)
Native American	1.0 (2)	1.6 (1)	.6 (1)
Other	1 (2)	0.0 (0)	1.3 (2)
Pacific Islander	0.05 (1)	1.6 (1)	0.0 (0)
Hispanic/Latino ethnicity	10.0 (22)	9.4 (6)	10.1 (16)
Education			
No high school diploma	2.7 (6)	4.7 (3)	1.9 (3)
High school diploma or equivalent (GED)	16.2 (36)	17.2 (11)	15.8 (25)
Some college credit, no degree	32.9 (73)	31.3 (20)	33.5 (53)
Trade/technical/vocational training after high school	4.1 (9)	6.3 (4)	3.2 (5)
Associate’s degree	9.9 (22)	6.3 (4)	11.4 (18)
Bachelor’s degree	27.9 (62)	25.0 (16)	29.1 (46)
Master’s degree	5.0 (11)	7.8 (5)	3.8 (6)
Professional/doctorate degree	1.4 (3)	1.6 (1)	1.3 (2)
Cigarette Smoking Characteristics			
Number of cigarettes smoked per day			
10 or fewer	73.4 (163)	73.4 (47)	73.4 (116)
11–20	21.6 (48)	25.0 (16)	20.3 (32)
21–30	3.6 (8)	1.6 (1)	4.4 (7)
More than 30	1.4 (3)	0.0 (0)	1.9 (3)
Time to first cigarette in the morning			
Within 5 min	40.5 (90)	40.6 (26)	40.5 (64)
6–30 min	16.6 (37)	15.6 (10)	17.1 (27)
31–60 min	27.5 (61)	32.8 (21)	25.3 (40)
After 60 min	15.3 (34)	10.9 (7)	17.1 (27)
Heaviness of Smoking Index			
Low dependence	51.3 (114)	50.0 (32)	51.6 (82)
Moderate dependence	46.8 (104)	43.8 (28)	45.6 (72)
High dependence	1.8 (4)	6.3 (4)	1.6 (4)
Number of days smoked in the past month (mean, SD)	21.3 (9.8)	21.2 (9.5)	21.4 (9.9)
Age at first cigarette (mean, SD)	15.4 (3.3)	16.2 (4.0)	15.1 (3.0)
Contemplation Ladder score (mean, SD)	4.8 (3.1)	5.1 (2.7)	4.7 (3.3)
Interest in quitting			
Yes	50.9 (113)	57.8 (37)	48.1 (76)
No	49.1 (109)	42.2 (27)	51.9 (82)
Interested in cutting down			
Yes	56.3 (125)	68.8 (44)	51.3 (81)
No	43.7 (97)	31.3 (20)	48.7 (77)

**Table 2:** Spearman’s rho correlation matrix for demographic variables, smoking characteristics, image variables, and switch point.

	Age	Sex	Income	Education	Cigarettes/Day	Image Is	Image Stop	Image Own Health	Image Others’ Health	Image Quit	Interest in Quitting	Quit Ladder	HSI	Have Children	Plan on Children	Switch Point
Age	--	--	--	--	--	--	--	--	--	--	--	--	--	--	--	--
Sex	0.01	--	--	--	--	--	--	--	--	--	--	--	--	--	--	--
Income	0.18 **	0.10	--	--	--	--	--	--	--	--	--	--	--	--	--	--
Education	0.16 *	0.08	0.40 **	--	--	--	--	--	--	--	--	--	--	--	--	--
Cigarettes/Day	0.07	0.11	0.04	-0.11	--	--	--	--	--	--	--	--	--	--	--	--
Image Is	−0.03	−0.08	−0.05	0.00	−0.11	--	--	--	--	--	--	--	--	--	--	--
Image Stop	−0.02	−0.15 *	0.03	0.07	−0.13	0.15 *	--	--	--	--	--	--	--	--	--	--
Image Own Health	−0.01	0.02	−0.02	0.05	−0.03	0.25 **	0.63 **	--	--	--	--	--	--	--	--	--
Image Others’ Health	0.02	−0.11	−0.08	0.02	−0.12	0.26 **	0.56 **	0.71 **	--	--	--	--	--	--	--	--
Image Quit	0.02	−0.07	0.01	0.06	−0.11	0.20 **	0.71 **	0.70**	0.69 **	--	--	--	--	--	--	--
Interest in Quitting	0.01	−0.02	−0.06	0.04	−0.04	0.10	0.28 **	0.30 **	0.23 **	0.40 **	--	--	--	--	--	--
Contemplation Ladder	0.00	0.03	−0.01	0.06	−0.14 *	0.11	0.35 **	0.32 **	0.31 **	0.48 **	0.72 **	--	--	--	--	--
HSI	0.10	0.14 *	0.06	−0.13	0.66 **	−0.06	−0.19 **	−0.08	−0.10	−0.15 *	−0.07	−0.22 **	--	--	--	--
Have Children	0.20 **	−0.27 **	0.08	0.00	−0.07	0.04	0.13	0.00	0.15 *	0.12	0.03	−0.02	0.04	--	--	--
Plan on Children	−0.25 **	0.05	0.08	0.04	0.14 *	−0.11	0.14 *	0.13	0.09	0.12	0.12	0.13	0.07	0.08	--	--
Switch Point	−0.05	−0.05	0.02	0.00	0.00	0.04	0.24 **	0.26^**^	0.20 **	0.29 **	0.07	0.02	−0.01	−0.01	0.03	--

* Correlation is significantly different from 0 at the 0.05 level (two-tailed). ** Correlation is significantly different from 0 at the 0.01 level (two-tailed). Image is = the message in this image is, see text for details. Image stop = image on my cigarette pack would stop me from having a cigarette, see text for details. Image own health = image on my cigarette pack would make me think about the risks of smoking to my own health, see text for details. Image others’ health = image on my cigarette pack would make me think about the risks of smoking to the health of others, see text for details. Image quit = image on my cigarette pack would make me think about quitting smoking, see text for details. HSI = heaviness of smoking index.

## Supplementary Materials

The following are available online at https://www.mdpi.com/1660-4601/16/18/3380/s1, Supplementary Figure S1: Screenshots of graphic warning labels (GWLs).
