# Supplementary figures and images for: Graphic Warning Labels Affect Hypothetical Cigarette Purchasing Behavior among Smokers Living with HIV
Source: Int J Environ Res Public Health. 2019 Sep 12;16(18):3380. doi: 10.3390/ijerph16183380 (PMC6765870; doi:10.3390/ijerph16183380)

**Supplemental Figure 1.** Screenshots of graphic warning labels (GWLs)

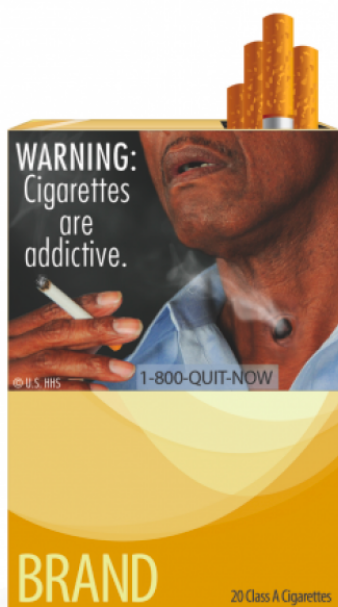

**\$7.00**

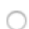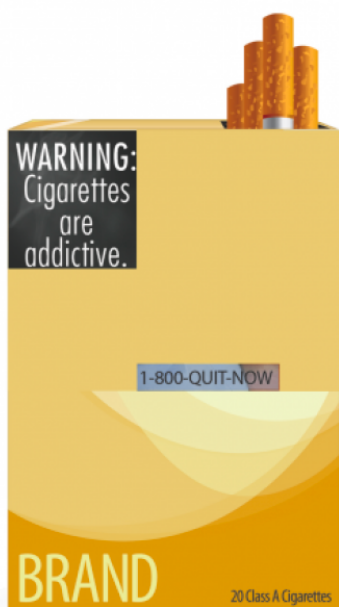

**\$3.50**

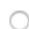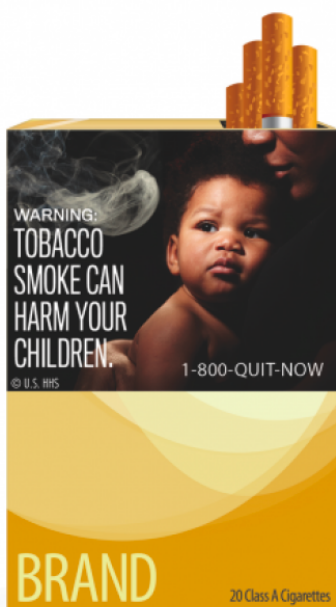

**\$7.00**

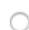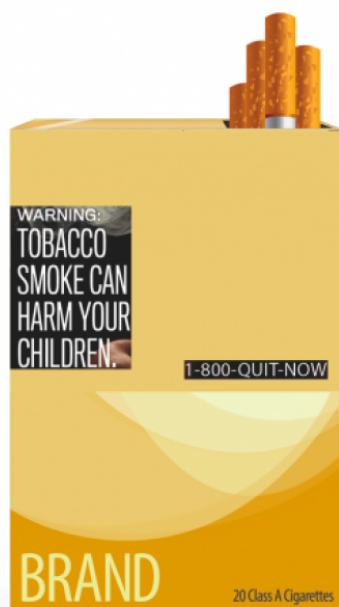

**\$3.50**

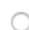

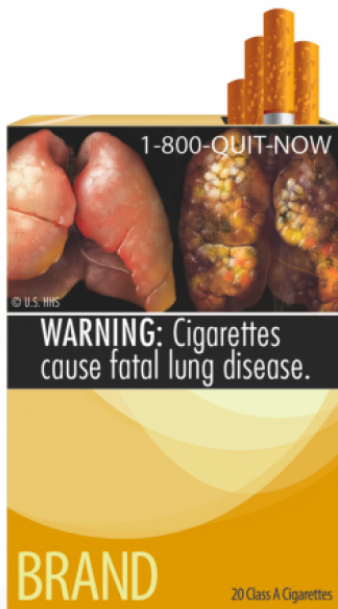

**\$7.00**

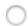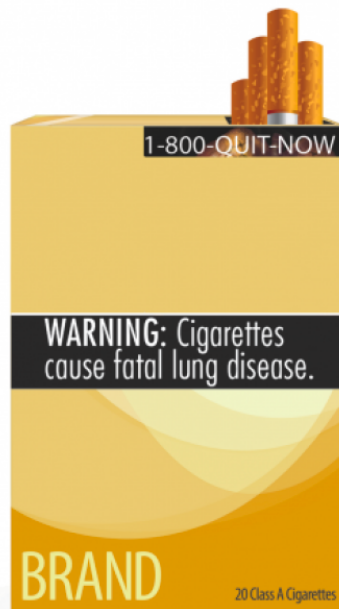

**\$3.50**

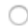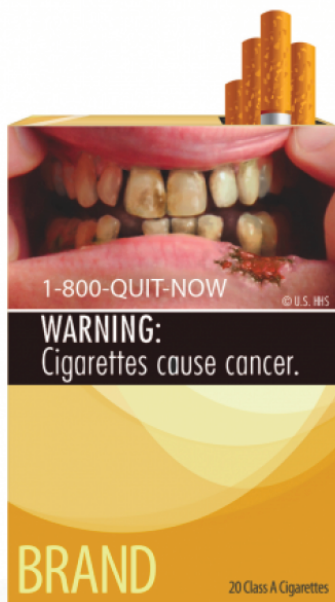

**\$7.00**

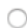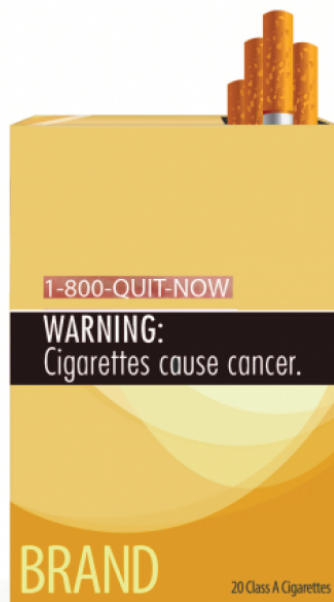

**\$3.50**

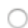

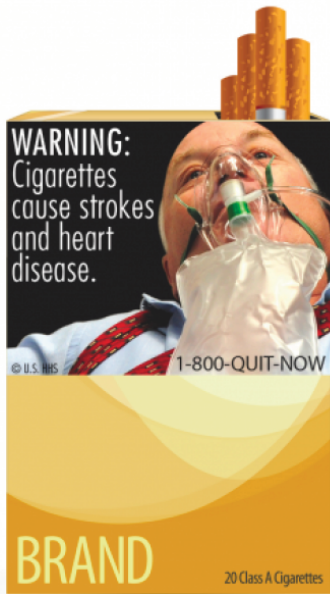

**\$7.00**

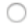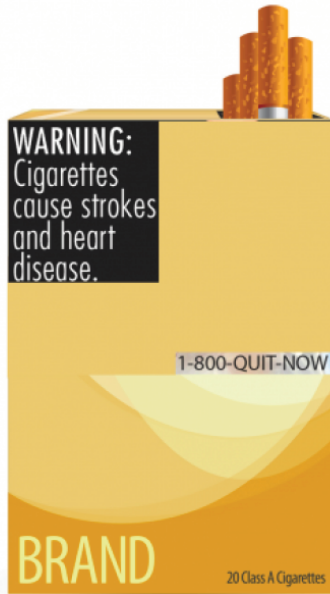

**\$3.50**

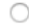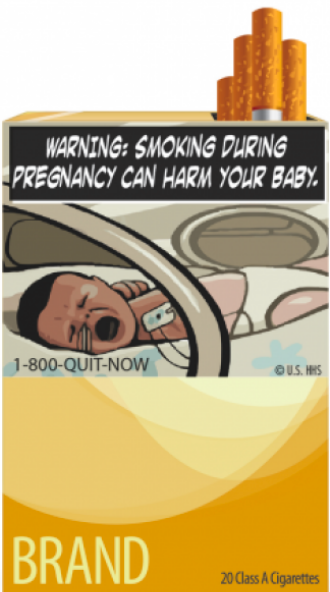

**\$7.00**

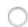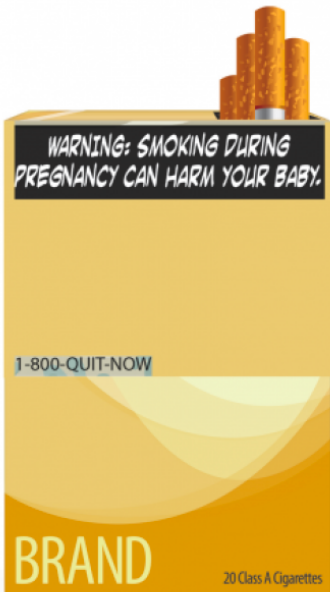

**\$3.50**

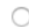

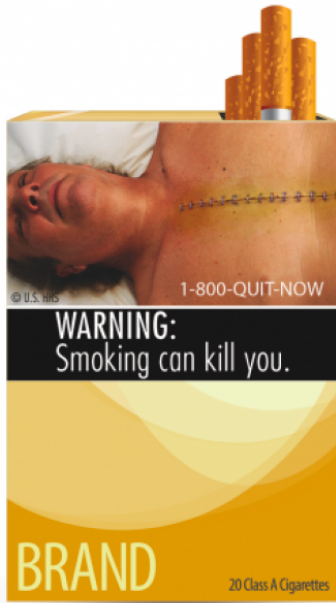

**\$7.00**

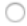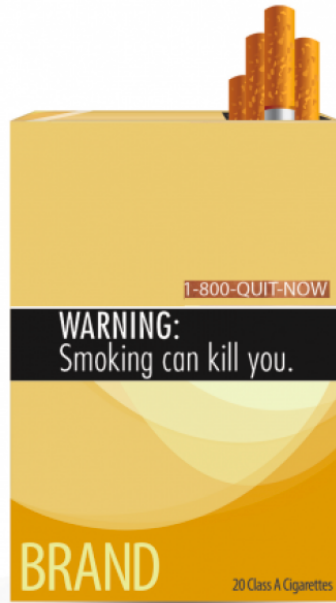

**\$3.50**

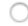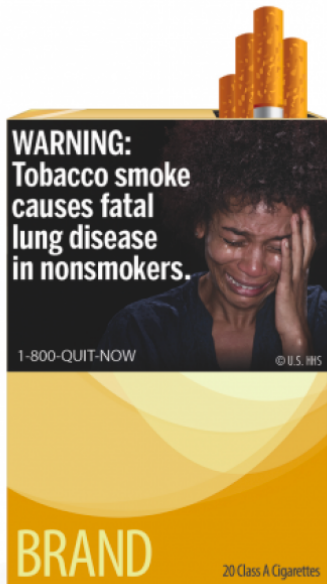

**\$7.00**

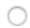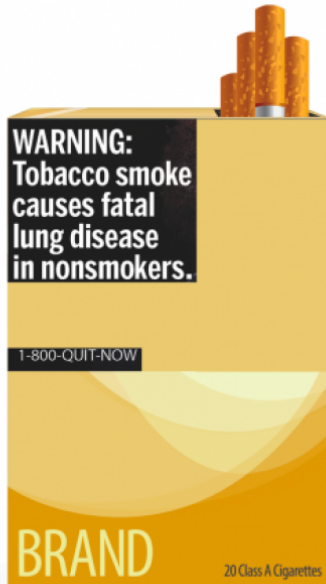

**\$3.50**

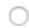

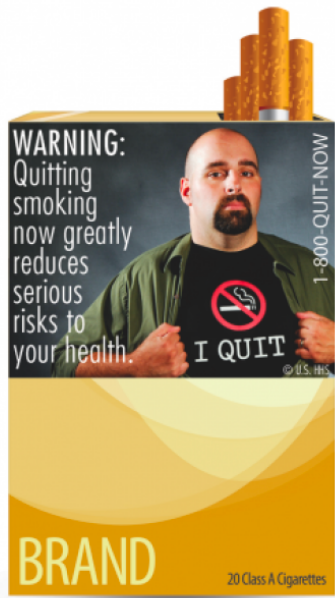

**\$7.00**

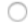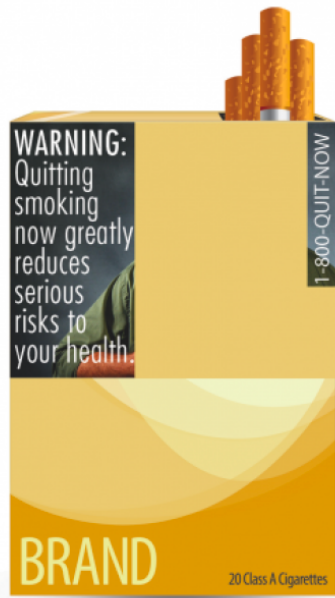

**\$3.50**

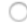

Supplement: Supplementary file 1 [file ijerph-16-03380-s001.pdf]
